# Molecular Characterization of Canine Rabies Virus, Mali, 2006–2013

**DOI:** 10.3201/eid2205.150470

**Published:** 2016-05

**Authors:** Abdallah Traoré, Evelyne Picard-Meyer, Stephanie Mauti, Melanie Biarnais, Oliver Balmer, Kassim Samaké, Badian Kamissoko, Saïdou Tembely, Amadou Sery, Abdel K. Traoré, Amy P. Coulibaly, Emmanuelle Robardet, Jakob Zinsstag, Florence Cliquet

**Affiliations:** Central Veterinary Laboratory, Bamako, Mali (A. Traoré, K. Samaké, B. Kamissoko, S. Tembely, A. Sery, A.P. Coulibaly);; French Agency for Food, Environmental, and Occupational Health and Safety, Malzéville, France (E. Picard-Meyer, M. Biarnais, E. Robardet, F. Cliquet);; Swiss Tropical and Public Health Institute, Basel (S. Mauti, O. Balmer, J. Zinsstag);; University of Basel, Basel, Switzerland (S. Mauti, O. Balmer, J. Zinsstag);; Faculty of Medicine and Odontostomatology, Bamako (A.K. Traoré)

**Keywords:** rabies virus, canine rabies virus, viruses, rabies, dogs, molecular analysis, Africa 2 lineage, Mali

## Abstract

We genetically characterized 32 canine rabies viruses isolated in Mali during 2006–2013 and identified 3 subgroups that belonged to the Africa 2 lineage. We also detected subgroup F rabies virus. This information should be useful for development of mass vaccination campaigns for dogs and eventual large-scale control programs in this country.

Rabies causes an estimated 70,000 human deaths annually worldwide, and >99% occur in developing countries, of which ≈43% occur in Africa, where rabies virus circulates in the dog population (*1*). A person bitten by a rabid dog, if not given postexposure prophylaxis, has an ≈5% (if bitten on the hand) to 70% (if bitten on the face) probability of showing development of clinical rabies (*2*). However, postexposure prophylaxis is often unavailable or unaffordable in many developing countries.

Numerous infectious diseases, including tuberculosis, malaria, dengue fever, and rabies, are present in Mali. The domestic dog is the major reservoir and vector of rabies in this country. Although disease surveillance is insufficient throughout Mali, the level of underreporting of rabies cases is unknown. Animal and human cases are recorded mainly in urban and suburban areas. Surveillance data reflect rabies mainly in Bamako (the capital of Mali; population 1.8 million), where rabies diagnostic testing is available.

A standard procedure is in place in Bamako for reporting of an animal bite. The bitten person should immediately contact the Division of Epidemiology, Prevention and Control of Diseases, which is part of the National Directorate of Health. Persons with suspected cases of rabies are referred to a specialized clinic (Lazaret Clinic) in Bamako. Dog owners are requested by the Division of Epidemiology, Prevention and Control of Diseases to bring their dogs to a veterinary clinic for a 15-day quarantine. Rabies diagnosis of suspect animals is made by the Central Veterinary Laboratory (CVL) in Bamako. A diagnosis of rabies in humans is based only on results of a clinical examination because of sociocultural reasons (*3*). In other cities in Mali, there are reference health centers, hospitals, and veterinary regional services for diagnosis (*4*).

During 2000–2013, samples from 468 animals showing clinical signs of rabies or to whom humans were exposed were submitted to the CVL for rabies testing by using the fluorescent antibody test (*5*). Of 468 animals analyzed, 447 (435 dogs, 4 cats, 4 cows, and 4 monkeys) showed positive results for rabies. Twenty-eight human cases of rabies were reported during 2007–2009 in Bamako, which indicated an incidence of 3.3 cases/1,000,000 persons/year despite 141 postexposure prophylaxis vaccinations/1,000,000 persons/year (*4*). Assuming a dog:human ratio in Bamako of 1:121, the annual incidence of rabies in dogs is ≈2.24 rabid dogs/1,000 dogs during the past 13 years, which is higher than that observed in N’Djaména, Chad (*6*), which borders Mali.

A total of 306 (45.0%; 95% CI 38%–52%) of 680 dogs were reported as being vaccinated against rabies at least once. However only 59 (19.3%) of the 306 dogs examined had a valid vaccination certificate (*4*).

In Bamako, an average of 1,470 persons are bitten by animals each year, of whom 1,427 (97.1%) are bitten by dogs (*3*). A total of 3,544 (60.3%) of 5,870 bitten persons are young adults, including 1,920 (32.73%) children <10 years of age. Men are bitten more often than women.

Four lineages (Africa 1–4) of rabies virus and several subgroups have been detected in Africa. All lineages include classical rabies virus species and vary by geographic area, virus evolution, and reservoir species (*7*,*8*). The most comprehensive study of western and central African rabies viruses included some isolates from Mali (*7*). The purpose of our study was to obtain more detailed information on genetic characteristics of rabies virus circulating in Mali and to clarify the geographic distribution and transboundary spread of this virus in the canine population in Mali.

## The Study

**Figure 1:**
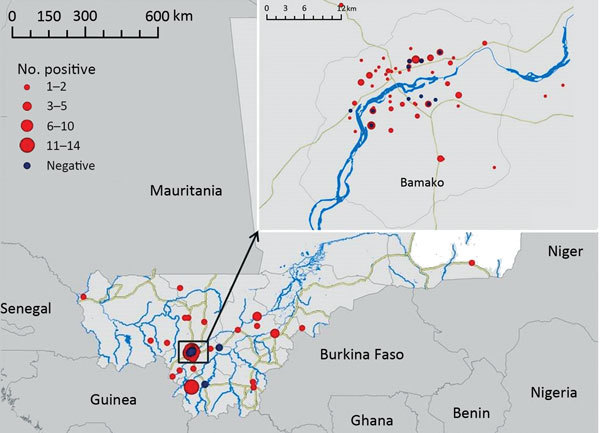
Locations of origin for 100 specimens analyzed in this study (95 with positive results and 5 with negative results) submitted for rabies virus diagnosis, Mali, 2002–2013. Inst shows closer view of the area near the capital city of Bamako.

During 2002–2013, a total of 468 specimens were submitted from various regions in Mali to the CVL for rabies diagnosis (Figure 1). Samples were tested by using the fluorescent antibody test (*5*) and stored at −20°C for further analyses. We selected 100 samples (95 with positive results and 5 with negative results) for further testing on the basis of their geographic origin.

Supernatants (100 μL) of suspensions (10% wt/vol) of dog brains were deposited on test paper cards, which stabilize nucleic acids. Virus RNA was extracted from stabilized samples by using the Iprep PureLink Virus Kit (Invitrogen, Paris, France) and subjected to partial nucleoprotein gene amplification of a conserved sequence (positions 55–660) (*9*). Virus RNA was tested by using a hemi-nested reverse transcription PCR (RT-PCR) and a real-time quantitative RT-PCR (*10*). After amplification, PCR products were sequenced in both directions by Beckman Coulter Genomics (Takeley, UK) and specific primers. A total of 32 stabilized samples showing positive results by hemi-nested RT-PCR and real-time, quantitative RT-PCR were used for phylogenetic analyses (Table 1).

**Table 1:** Characteristics of 32 rabies virus samples from dogs, Mali, 2006–2013*

Virus	GenBank accession no.	Sample ID no.	Region	Quantitative RT-PCR C_t_	Subgroup of Africa 2 lineage
RV01	KP976113	420/2006	Bamako	28.51	G
RV04	KP976114	345/2007	Bamako	30.19	H
RV05	KP976130	352/2007	Bamako	25.35	G
RV06	KP976125	58/2008	Bamako	24.09	G
RV09	KP976126	146/2008	Bamako	27.51	G
RV10	KP976122	154/2008	Ségou	25.75	G
RV11	KP976124	167/2008	Koulikoro	23.97	G
**RV14**	NA	259/2008	Bamako	31.59	H
RV15	KP976123	261/2008	Ségou	26.14	G
**RV19**	NA	530/2008	Bamako	27.85	G
RV20	KP976117	003/2009	Bamako	32.82	H
**RV22**	NA	69/2009	Bamako	26.15	H
**RV27**	NA	118/2009	Bamako	32.30	H
RV44	KP976129	587/2009	Bamako	26.22	G
**RV50**	NA	19/11/2010	Bamako	27.90	G
**RV51**	NA	42/2010	Bamako	28.59	G
**RV56**	NA	171/2010	Koulikoro	22.20	G
RV57	KP976121	176/2010	Gao	21.90	F
**RV60**	NA	221/2010	Bamako	24.60	H
**RV67**	NA	603/2010	Bamako	21.30	H
**RV68**	NA	137/2011	Bamako	20.80	H
RV70	KP976119	149/2011	Bamako	21.70	H
**RV79**	NA	339/2011	Bamako	24.90	G
RV81	KP976127	357/2011	Bamako	34,20	G
**RV84**	NA	480/2011	Bamako	21.20	G
RV87	KP976116	612/2011	Bamako	22.50	H
**RV88**	KP976120	628/2011	Koulikoro	21.70	H
**RV89**	NA	674/2011	Bamako	20.20	H
RV90	KP976118	688/2011	Bamako	30.80	H
RV93	KP976115	223/2012	Bamako	23.20	H
**RV95**	NA	366/2012	Bamako	21.00	G
RV96	KP976128	100/2013	Bamako	29.00	G
*A fluorescent antibody test was conducted as described by Dean et al. (*5*). For each tested sample, test paper was impregnated with 100 μL of 10% brain suspension and subjected to molecular biological analysis. Of 100 samples tested, 32 showed positive results by this test. A conventional hemi-nested reverse transcription PCR (RT-PCR) was performed with rabies virus primers JW12–JW6 as described (*9*). All samples showed positive results by this test. A quantitative RT-PCR was performed with rabies primers JW12–N165-146 (*10*). This PCR detected >100 RNA copies/µL. The coefficient of determination (R^2^) was 0.999, the Y intercept was of 36.65, and efficiency was 99%. Samples in bold (n = 15) had duplicate sequences and were not subjected to phylogenetic analysis. ID, identification; C_t_, cycle threshold; NA, not available.					

**Figure 2:**
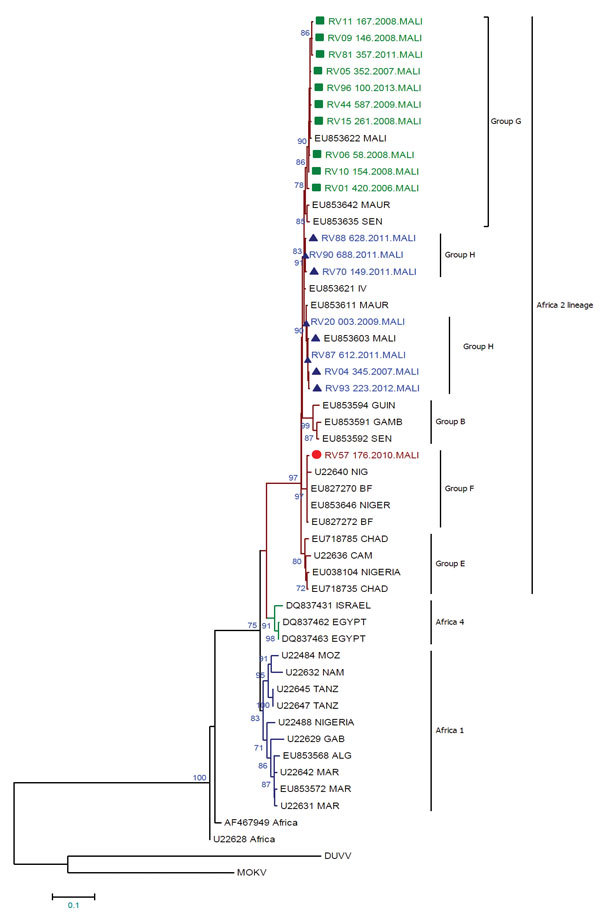
Maximum-likelihood phylogenetic tree based on a 564-nt sequence of nucleoprotein genes of 18 rabies virus sequences from Mali, 2002–2013, and representative sequences from Mali (n = 2), northern Africa (n = 6), South Africa (n = 2), West Africa (n = 32), and central Africa (n = 5). Sequences obtained in this study are identified in green, blue, and red. Green squares indicate genotype G, blue triangles indicate genotype H, and red circles indicate genotype F. The tree is rooted with 2 bat isolates used as outgroups Duvenhage virus (DUVV) (U22848) and Mokola virus (MOKV) (U22843). Bootstrap values (100 replicates) >70% are shown next to nodes. Alg, Algeria; BF, Burkina Faso; Cam, Cameroon; Gab, Gabon; Gamb, Gambia; Guin, Guinea; Maur, Mauritania; Mar, Morocco; Moz, Mozambique; Nig, Niger; Sen, Senegal; Tanz, Tanzania. Scale bar indicates nucleotide substitutions per site.

We constructed a maximum-likelihood phylogenetic tree (Figure 2) that excluded 15 duplicate sequences (Table 2) by using MEGA version 6 software (*11*). We also constructed a maximum-parsimony haplotype network by using TCS version 1.21 software (*12*).

**Table 2:** Characteristics of representative nucleoprotein gene sequences for rabies virus isolates, Mali, 2006–2013*

Isolate	Haplotype	Identical sequences (−546 nt of the N gene)	Phylogroup
RV09	2	RV50, RV56, RV51, RV19, RV79	G (Africa 2)
RV96	6	RV84, RV95	G (Africa 2)
RV90	11	RV67, RV60, RV68, RV88, RV89, RV22	H (Africa 2)
RV87	14	RV14, RV27	H (Africa 2)

*All identical sequences have 100% nucleoprotein (N) gene identity on the basis of 546 nt (positions 71–618) compared with the reference isolate. RV, rabies virus.

We analyzed phylogenetic relationships between 18 partial nucleoprotein gene sequences and 31 representative sequences of Africa lineages of rabies virus. This analysis (Technical Appendix Figure 1) showed that all samples that belonged to the Africa 2 lineage were widely distributed in western and central Africa (*7*), including Mali and neighboring countries (Mauritania, Guinea, Senegal, Niger, Nigeria, Côte d’Ivoire, and Burkina Faso).

We found <2.1% divergence between all isolate sequences. For 17 haplotypes, 10 sequences were identified as belonging to subgroup G; this subgroup also included 3 sequences from Mali, Mauritania, and Senegal. Seven sequences (forming 6 haplotypes; RV88 was identical to RV90) belonged to subgroup H, which contained representative sequences from Côte d’Ivoire, Mauritania and Mali. One sequence from Mali (isolate RV57) belonged to subgroup F, which was similar to sequences from neighboring countries (Niger and Burkina Faso). Our data indicate that subgroup H might contain 2 distinct groups (Technical Appendix Figure 2).

Analysis of the nucleoprotein gene identified canine rabies subgroups G and H in Mali, as reported (*7*), and subgroup F, which was found throughout Burkina Faso and Niger (*8*). Subgroup G circulates in Mauritania, Burkina Faso, and Senegal. Subgroup H contains viruses from Mauritania, Mali, Burkina Faso, and Côte d’Ivoire. The RV57 isolate included in subgroup F was isolated from a rabid dog at the border with Niger in 2010. Strong nucleotide identity (99.6%) was shown between RV57 and the strain isolated in Niger in 2010 (Genbank accession no. EU853646).

## Conclusions

We identified 3 subgroups of the Africa 2 lineage of rabies virus in Mali. The presence of subgroup F could be explained by the movement of rabid animals across country borders. Previous studies reported rabies virus transmission by human-mediated animal movements (*13*,*14*). The information we obtained in this study should be useful for development of mass vaccination campaigns for dogs and eventual large-scale control programs in this country.

**Technical Appendix.** Additional information on molecular characterization of canine rabies virus, Mali, 2006–2013.
