# Supplementary material for: Molecular Characterization of Canine Rabies Virus, Mali, 2006–2013
Source: Emerg Infect Dis. 2016 May;22(5):866–70. doi: 10.3201/eid2205.150470 (PMC4861505; doi:10.3201/eid2205.150470)
Supplement: Supplementary file 1 — Technical Appendix. Additional information on molecular characterization of canine rabies virus, Mali, 2006–2013. [file 15-0470-Techapp-s1.pdf]

Article DOI: http://dx.doi.org/10.3201/eid2206,150470

Molecular Characterization of Canine Rabies Virus, Mali, 2006–2013

Technical Appendix

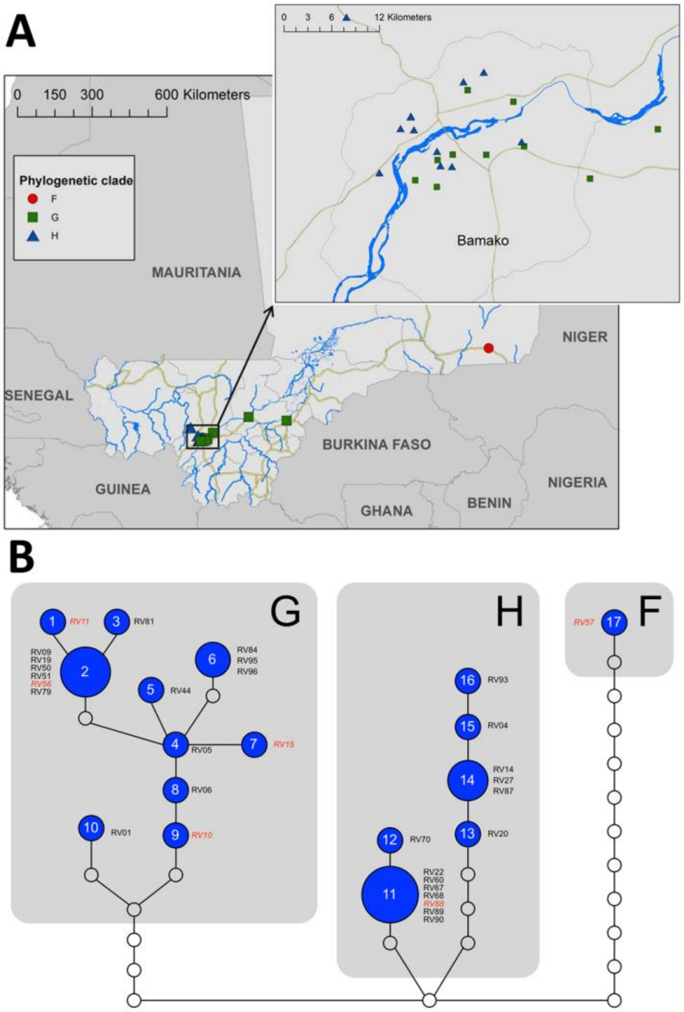

Technical Appendix Figure. A) Locations and phylogenetic clades for 32 rabies virus isolates obtained in Mali during 2006–2013. Clades were identified on the basis of results of gene phylogenetic analysis of nucleoprotein gene sequences. B) Maximum-parsimony haplotype network of 32 rabies virus isolates based on 564-nt nucleoprotein gene sequences. Numbers 1–17 indicate haplotype ID, dot size indicates number of samples per haplotype. Each line indicates 1 mutation, empty dots indicate haplotypes not sampled, Sequence codes in red and italics (Table 1 in main text) indicate samples not from the Bamako region.
